# The lifestyle and nutritional factors for dry eye disease in depression population: a retrospective case–control study

**DOI:** 10.3389/fmed.2024.1376938

**Published:** 2024-09-10

**Authors:** Chia-Yi Lee, Shun-Fa Yang, Ie-Bin Lian, Yu-Ling Chang, Yan-Ni Jhan, Chao-Kai Chang

**Affiliations:** ^1^Institute of Medicine, Chung Shan Medical University, Taichung, Taiwan; ^2^Nobel Eye Institute, Taipei, Taiwan; ^3^Department of Ophthalmology, Jen-Ai Hospital Dali Branch, Taichung, Taiwan; ^4^Department of Medical Research, Chung Shan Medical University Hospital, Taichung, Taiwan; ^5^Institute of Statistical and Information Science, National Changhua University of Education, Changhua, Taiwan; ^6^Department of Medical Education, Cathay General Hospital, Taipei, Taiwan; ^7^Department of Optometry, Da-Yeh University, Chunghua, Taiwan

**Keywords:** dry eye disease, depression, coffee, tea, exercise

## Abstract

**Background:**

We aim to evaluate lifestyle and nutritional factors that lead to dry eye disease (DED) in a depressed population using data from the Taiwan BioBank (TWB).

**Methods:**

A retrospective case–control study was conducted, and patients with depression based on a questionnaire were selected as the depression group. Each patient in the depression group was matched by age and sex to two individuals without depression, and a total of 3,754 and 7,508 patients constituted the depression and non-depression groups, respectively. Based on the questionnaire, the primary outcome was the presence of DED. Additionally, the chi-square test and interaction test were applied to survey the effect of lifestyle and nutritional factors on DED in the depression and non-depression groups.

**Results:**

There were 822 (21.90%) and 958 (12.76%) DED patients in the depression and non-depression groups, respectively, and the incidence of DED was significantly higher in the depression group (*p* < 0.001). In terms of lifestyle and nutritional factors in the depression population, a higher rate of chronic pain and a sedentary lifestyle were observed than in the patients with depression without DED (both *p* < 0.05). According to the interaction test, the chronic pain (*p* = 0.0227) and sedentary lifestyle (*p* = 0.0002) were significant risk factors for DED presence in the depression group than in the non-depression group, while the persistent coffee consumption (*p* = 0.0005) and tea consumption (*p* = 0.0003) were significant protective factors for the DED exclusively for the depression group and not for the non-depression group.

**Conclusion:**

The depression population could be significantly benefited from physical activity, coffee intake and tea intake regarding DED development compared to the general population.

## 1 Introduction

Dry eye disease (DED) is one of the most prevalent ophthalmic diseases, with the highest occurrence in Southeast Asia (1). Although many DED cases are not severe, advanced-stage DED can occur in one of five Americans diagnosed with DED (2). Currently, the management of DED includes artificial tears, warm compression, intense pulsed light therapy, and lipid-containing eye drops (3–5). Additionally, certain diseases, such as rheumatoid arthritis, Sjogren’s syndrome, systemic lupus erythematosus, and medications for autoimmune diseases, including methotrexate and cyclophosphamide, are associated with the development of DED, which should be examined when addressing DED (2).

In addition to the aforementioned systemic diseases, DED occurrence correlates with lifestyle and nutritional factors. A previous study showed that increased digital screen exposure is a risk factor for DED development (6). Moreover, individuals using a visual display terminal (VDT) device in their work may experience a more prevalent DED incidence ranging from 26 to 70% (7). Another study implied that ex-smokers experienced more DED (8), and that decreased sleeping hours per day was similarly related to the DED rate (9). Interestingly, increasing physical activity can retard the incidence of DED (10). However, whether modified lifestyle and nutritional factors interact with other DED-related factors remains unclear.

Depression is an affective disorder strongly associated with DED (11). In a previous study, DED was more common in individuals with depressive disorders (8), and another population-based study in a Chinese population similarly demonstrated a prominent association between depression and DED (12). Moreover, antidepressants can contribute to subsequent DED in patients with depressive disorders (13). Nevertheless, there has been a rare report on whether any specific lifestyle and nutritional factors cause DED in depression. Since lifestyle and nutritional factors could potentially lead to DED, preventing depression in patients with double risk factors cannot be disregarded. Therefore, this current study aimed to evaluate the lifestyle and nutritional factors for DED event in the depression population based on the Taiwan Biobank (TWB) database, and the effect of age and gender were included in the multivariable analysis.

## 2 Materials and methods

### 2.1 Data source

The TWB is a database constructed by the Academia Sinica of Taiwan. Taiwan has over 30 TWB data collection stations dispersed throughout its northern, central, southern, and eastern regions. From 2012 to 2022, approximately 172,078 individuals joined the TWB project, and the sample of each participant was obtained after explanation and signing the written informed consent provided by the TWB. The data available in the TWB included age, sex, place of residence, occupation, socioeconomic status, personal history, lifestyle habits, nutrition usage, and medical history. All the above information was obtained using a questionnaire. Body information, laboratory data, and genome analyses were also available in the TWB database.

### 2.2 Participant selection

**Figure 1:**
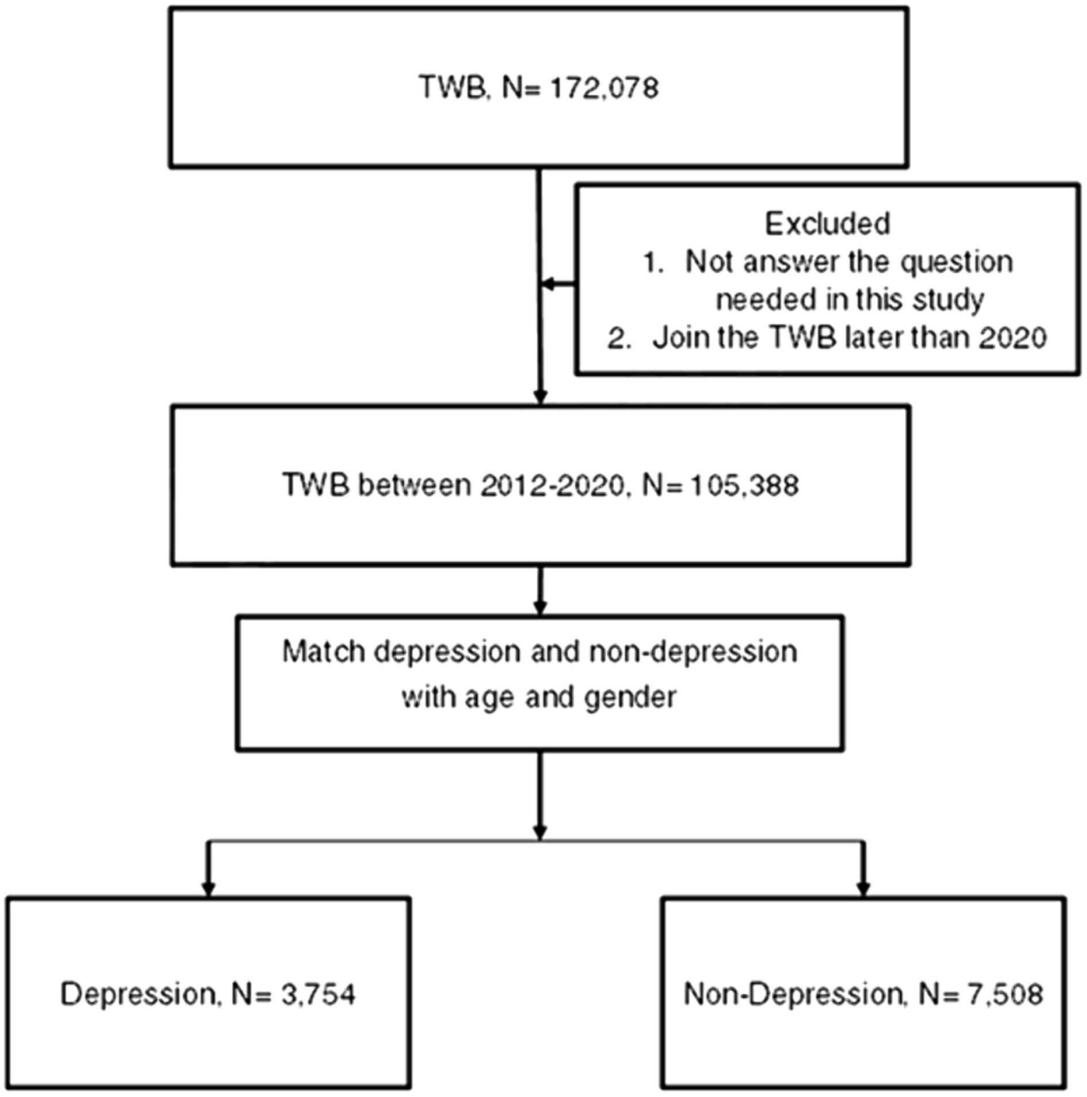
Flowchart of patient selection. TWB, Taiwan Biobank; N, number.

A retrospective case–control study was conducted, and patients were considered a member of the depression population if their TWB records indicated the following information: (1) Han ethnicity, (2) depression diagnosis according to the questionnaire filled by each participant, (3) the year of depression diagnosis presented in the questionnaire filled by each patient, and (4) enrollment data ranging from 2012 to 2020. For comparison, each participant in the depression group was matched by age and sex to two individuals who did not have a history of depression diagnosis but belonged to the Han ethnicity and enrolled in the TWB between 2012 and 2020, and the latter population constituted the control group. Notably, we matched the two groups according to age and sex because of the prominent effect of age and sex on DED (2). For each participant in the depression group, we performed 1-to-2 matching with participants in the non-depression group based on age and sex. This matching was conducted using the PROC PSMATCH procedure in SAS 9.4. In this procedure the propensity score (PS) was predicted probability of depression estimated by age and gender, and then the “greedy” algorithm was applied such that each depression participant (in random order) was matched with two non-depression participants with two nearest PS within the specified caliper. For our study, a caliper of 0.2 was used. Additionally, patients who did not answer the questions that were required to be collected and analyzed in this study were excluded. Following the selection process, 3,754 and 7,508 individuals were categorized as depression and non-depression groups, respectively. Moreover, the education level, marital status, and place of residence of the study population were obtained from the same questionnaire, and the patients who did not answer the question required in the questionnaire were discarded. The flowchart of the patient selection process is shown in Figure 1.

### 2.3 Outcome measurement and covariates

The primary outcome of this study was the history of DED event, which was defined as follows: (1) diagnosis of DED according to the questionnaire filled by each participant and (2) the year of DED diagnosis presented in the questionnaire filled by each patient. Additionally, we collected certain lifestyle and nutritional factors from the questionnaire obtained from the participants and enrolled them in subsequent analyses. The lifestyle and nutritional covariates obtained for this study included VDT use, persistent alcohol drinking, persistent cigarette smoking, persistent betelnut chewing, sedentary lifestyles, oily and salty diet, vegan diet, persistent coffee consumption and persistnet tea consumption. Furthermore, the presence of a history of chronic pain, which was defined by the questionnaire as pain at any site for over 3 months, was collected. For more details of the lifestyle and nutritional factors, the VDT users were defined as those engaged in the wholesale industry, retail business, international trade, communication industry, financial industry, insurance business, real estate industry, law practice, investment industry, public administration, education industry, mass communication, medical practice, cultural industry, and international institutions who would frequently use the computer or cell phone during work. Persistent alcohol consumption, cigarette smoking, betelnut chewing, coffee consumption and persistent tea consumption were defined as the current use of the mentioned substances which has persisted for at least 5 years. A sedentary lifestyle was regarded as a routine with no sports habits, while the oily and salty diet habits denoted high-fat and high-salt diet responses in more than four of eight items concerning diet habits. The vegan diet referred to a vegetarian diet status for more than 5 years.

### 2.4 Statistical analysis

All analyses were performed using SAS version 9.4 (SAS Institute Inc., Cary, NC, United States). Descriptive analyses were performed to present the basic characteristics of the two groups. The independent T-test was applied to analyze the age at enrollment between the two groups, while the chi-square test was used for the analysis of gender, education level, marital status, place of residence, and lifestyle and nutritional factors between the two groups. The incidence of DED between the depression and non-depression groups was also analyzed using the chi-square test. Furthermore, we divided the depression group into those with and without DED, and the chi-square test was used to assess the distribution of each lifestyle and nutritional factor between the two subgroups. Notably, the ratio of family history of depression and persistent depression, defined as an interval of depression greater than 5 years, was also evaluated in this step. Additionally, interaction test was applied to compare the effect of each variable on the presence of DED between the two groups. Besides, we categorized the depression group into patients with depression interval less or equal to 10 years and patients with depression period for more than 10 years, and the logistic regression was used for producing the adjusted odds ratio (aOR) with correlating 95% confidence interval (CI) and *p* value. A *p* value defined as *p* < 0.05 was considered to have statistical significance, and a *p* value less than 0.0001 were depicted as *p* < 0.0001 in the current study.

## 3 Results

### 3.1 Basic characteristics of the depression group and non-depression group

The basic characteristics of the study population are shown in Table 1, and statistical analysis was adopted for the comparison of the features between the two groups to examine the homogeneity. The mean age and male ratios were 51.34-years old and 23.51%, respectively, in both groups due to the matching process (*p* = 1.000). Conversely, the non-depression group having higher education levels and more married individuals compared to the depression group (both *p* < 0.0001). For the lifestyle and nutritional covariates, a higher ratio of chronic pain, persistent cigarette smoking, and VDT users was observed in the depression group (all *p* < 0.05).

**Table 1:** Characteristics of the study population.

Variable (%)	Depression (*N* = 3,754)	Non-depression (*N* = 7,508)	*p* value
Age (mean ± SD)	51.34 ± 0.79	51.34 ± 0.79	1.000
Gender (male)	883 (23.52)	1,766 (23.52)	1.000
Education			<0.001*
Elementary school	236 (6.29)	533 (7.10)	
High school	1,717 (45.74)	3,067 (40.85)	
University and above	1,801 (47.97)	3,908 (52.05)	
Marriage			<0.0001*
Unmarried	517 (13.77)	882 (11.75)	
Married	2,276 (60.63)	5,550 (73.92)	
Divorced	690 (18.38)	655 (8.72)	
Widowed	271 (7.22)	421 (5.61)	
Place of residence			0.1659
Northern	1,157 (30.82)	2,411 (32.11)	
Central	900 (23.97)	1833 (24.41)	
Southern	1,455 (38.76)	2,776 (36.97)	
Eastern	225 (5.99)	432 (5.75)	
Outlying Islands	19 (0.46)	58 (0.76)	
Chronic pain	651 (17.34)	1,038 (13.83)	<0.0001*
Persistent alcohol drinking	141 (3.76)	268 (3.57)	0.6180
Persistent cigarette smoking	434 (11.56)	512 (6.82)	<0.0001*
Persistent betelnut chewing	145 (3.86)	234 (3.12)	0.0385*
Sedentary lifestyle	1,628 (43.37)	3,216 (42.83)	0.6737
Oily and salty diet habit	87 (2.32)	170 (2.26)	0.8583
Vegan	34 (0.91)	63 (0.84)	0.7184
VDT user	370 (9.86)	624 (8.31)	0.0064*
Persistent coffee consumption	295 (7.86)	598 (7.96)	0.0939
Persistent tea consumption	215 (5.73)	471 (6.27)	0.0132*

N, number; SD, standard deviation; VDT, visual display terminal.

Independent *T*-test was applied to analyze the age at enrollment between the two groups while the chi-square test was used for the analysis of the rest covariates between the two groups.

*Denotes significant difference between the two groups.

### 3.2 Incidence of DED in the two groups

There were 822 (21.90%) and 958 (12.76%) DED patients in the depression and non-depression groups, respectively (Table 2). To compare the overall incidence of DED between the two groups, the rate of DED was 21.90% in the depression group, which was significantly higher than the 12.76% in the non-depression group (*p* < 0.001).

**Table 2:** Incidence of dry eye between the depression and non-depression groups.

Ratio	DED (%)	Non-DED (%)	Total	*p* value
Depression	822 (21.90)	2,932 (78.10)	3,754	
Non-depression	958 (12.76)	6,550 (87.24)	7,508	
Total	1,780	9,482	11,262	<0.001*

DED, dry eye disease.

Chi-square test was used for the analysis of DED incidence between the two groups.

*Denotes significant difference between the two groups.

### 3.3 Subgroup analysis for the lifestyle and nutritional factors of DED in depression population

Regarding the subgroup analysis in the depression population for evaluating possible factors related to DED event, the patients with DED were older and had a higher female ratio than the individuals without DED (both *p* < 0.0001) (Table 3). The mean age of depression patients with DED was 55.21 ± 0.63 years, which is about 5 years older than the mean age of depression patients without DED (50.25 ± 0.37 years old). The similar tendency was also found in the non-depression individuals with DED compared to the non-depression individuals without DED (55.89 ± 0.44 years versus 50.67 ± 0.46 years, respectively). Regarding lifestyle and nutritional factors, depression patients with DED demonstrated a higher rate of chronic pain and a sedentary lifestyle than those without DED (both *p* < 0.05). In contrast, the incidences of persistent alcohol drinking, persistent betelnut chewing, and oily and salty diet habits were significantly lower in the patients in the depression with DED subgroup (all *p* < 0.05) (Table 3). For the better understanding, betel nut chewing is a habit in which betel nuts are chewed together with the slaked lime and the betel leaves for the narcotic and stimulant effects, which is prevalent in Southeast Asia, Taiwan, southern China and South Asia. The betel nut chewing is addictive and lead to major health issues, which including oral cancers, esophageal cancers, and cardiovascular disorders. About the results of interaction test, both the chronic pain (*p* = 0.0227) and sedentary lifestyle (*p* = 0.0002) were significant risk factors for DED presence in the depression group than in the non-depression group, while the persistent coffee consumption (*p* = 0.0005) and persistent tea consumption (*p* = 0.0003) were significant protective factors for the DED event in the depression group than in the non-depression group (Table 3).

**Table 3:** The lifestyle and nutritional factor and dry eye event in the depression population compared to non-depression population.

Variable (%)	Depression group		Non-depression group		*p* value^#^	Interaction *p*
	DED (*N* = 822)	Non-DED (*N* = 2,932)	DED (*N* = 958)	Non-DED (*N* = 6,550)		
Age (mean ± SD)	55.21 ± 0.63	50.25 ± 0.37	55.89 ± 0.44	50.67 ± 0.46	<0.0001*	0.8765
Gender (male)	109 (13.26)	774 (26.40)	143 (14.93)	1,613 (24.78)	<0.0001*	0.9024
Family history of depression	120 (14.60)	392 (13.37)	N/A	N/A	0.3643	N/A
Persistent depression	412 (50.43)	1,511 (52.12)	N/A	N/A	0.3923	N/A
VDT user	85 (10.34)	285 (9.72)	81 (8.46)	543 (8.29)	0.5980	0.0740
Chronic pain	185 (22.51)	466 (15.89)	147 (15.34)	891 (13.60)	0.0122*	0.0227*
Persistent alcohol drinking	18 (2.19)	123 (4.20)	27 (2.82)	241 (3.68)	0.0075*	0.0692
Persistent cigarette smoking	60 (7.30)	374 (12.76)	52 (5.43)	460 (7.02)	<0.0001*	0.1493
Persistent betelnut chewing	15 (1.82)	130 (4.43)	18 (1.88)	216 (3.30)	0.0006*	0.5581
Sedentary lifestyle	409 (49.76)	1,218 (41.54)	434 (45.30)	2,782 (42.47)	<0.0001*	0.0002*
Oily and salty diet habit	11 (1.34)	76 (2.59)	16 (1.67)	154 (2.35)	0.0347*	0.2354
Vegan	6 (0.73)	28 (0.95)	7 (0.73)	56 (0.85)	0.5472	0.7418
Persistent coffee consumption	42 (5.11)	253 (8.63)	73 (7.62)	525 (8.02)	<0.0001*	0.0005*
Persistent tea consumption	31 (3.77)	184 (6.28)	59 (6.16)	412 (6.29)	<0.0001*	0.0003*

DED, dry eye disease. N, number; SD, standard deviation; VDT, visual display terminal. N/A, not applicable; Interaction *p*, comparison of *p* value for each variable between the depression group and non-depression group.

Chi-square test was used to compare the distribution of each lifestyle and nutritional factor between the DED and non-DED subgroups, and interaction test was applied to compare the relationship of each variable on the DED event between the depression and non-depression groups.

*Denotes significant difference between the two groups.

^#Between the DED and non-DED population of the depression group.^

### 3.4 Association of disease interval of depression on the DED events

For the comparison between individuals with different disease interval of depression to find the potential correlation between depression interval and DED event, there were 604 individuals with depression less than 10 years diagnosed with DED while another 218 patients with depression more than 10 years diagnosed with DED. The percentage of DED was higher in the patients with depression more than 10 years but without statistical significance (*p* = 0.1038) (Table 4).

**Table 4:** The distribution of dry eye in persistent gout patients with different disease period.

Interval	DED (%)	Non-DED (%)	Total	aOR (95% CI)	*p* value
Depression ≤10 years	604 (21.07)	2,263 (78.93)	2,867		
Depression >10 years	218 (24.58)	669 (75.42)	887		
Total	822	2,932	3,754	1.351 (0.747–2.659)	0.1038

DED, dry eye disease. aOR: adjusted odds ratio; CI, confidence interval.

Logistic regression was used to compare the DED incidence between depression populations with different course of depression.

## 4 Discussion

In this study, the depression group showed a significantly higher ratio of DED than the non-depression group. Moreover, patients with depression who experienced chronic pain and a sedentary lifestyle demonstrated a higher possibility of developing DED than those with depression without the two lifestyle and nutritional factors. Conversely, the percentage of persistent coffee consumption and persistent tea consumption was significantly lower in patients with depression and DED than those depression populations without DED.

Several studies have explored the relationship between depression and DED, and a strong association has been established between these two diseases (8, 11–13). The adjusted OR of DED significantly increased in patients with anxiety and depressive disorders, according to a recent large-scale study (14), and another study revealed that DED is commonly diagnosed in patients with new-onset anxiety and depression (15). In contrast, the DED population is at a higher risk of experiencing depression and has more depression and anxiety episodes than the general population (11). Notably, as for the potential mechanism of the correlation between DED and depression, the persistent ocular irritation and dryness in DED may contribute to subsequent depressive disorders (16). Additionally, persistent DED causes a visual disturbance, whereas impaired vision is another predisposing factor for depressive disorders (17).

Moreover, medication for depression, such as using selective serotonin reuptake inhibitors for depression disorders, aggravates depression-associated DED, which results from the increase in tear serotonin levels and ocular surface inflammation via the NF-κB pathway (18). Contrarily, depression is associated with some chronic inflammatory diseases, such as chronic pain and rheumatic arthritis (19, 20), while chronic inflammatory reactions can contribute to the development and progression of DED (21). Similarly, the presence of specific lifestyle and nutritional factors could cause stress in the human body, and stress could be a prominent factor in the occurrence of DED (22). Consequently, we speculated that while certain lifestyle and nutritional factors may not influence the ocular surface in the normal population, they may alter the possibility of DED in high-risk populations, including those with co-existing depressive disorder. Notably, this concept is supported by the findings of the present study.

This study demonstrated a higher incidence of DED in patients with depression than in the non-depression population which positive correlated to the period of depression disease. This finding corresponded with previous reports that DED is associated with depressive disorder (12). In further analysis of this study, we observed that patients with depression and DED showed a higher incidence of co-existing chronic pain and a sedentary lifestyle than those with depression without DED. To the best of our knowledge, few studies have discussed this issue. According to previous studies that share similar biological pathways and neurotransmitters, chronic pain is a known risk factor for both depression and DED (19, 23). For the possible etiology, the inflammatory reaction in chronic pain may aggregate to the disease course of DED (23), and the persistent discomfort may be associated with depressive mood (19). Since depression has a higher chance of developing DED (8), the additional inflammation from chronic pain could trigger the development of DED in such high-risk populations much earlier than in the general population. Conversely, a sedentary lifestyle refers to the absence of sports or other physical activities during daily life. In a previous study, an increase in physical activity for 2 months improved the DED status, mainly resulting from the reduction of subjective symptoms (10). Additionally, to subjective symptoms, physical activity or exercise increases tear film volume (24), and a previous meta-analysis revealed a promising effect of exercise on depression control (25). Positive emotions due to physical activity may ameliorate both the symptoms of DED and depressive disorders because DED is related to mental health problems (26), thus, patients without such protective factors are more vulnerable to DED episodes. On the other hand, both the persistent coffee and tea consumptions correlated to the absent of DED more prominent in the depression than the non-depression population. This may also be a preliminary experience up to now. The coffee intake was not a risk factor for the development of DED (27), and the extracts from tea tree can retard the signs and symptoms of meibomian gland dysfunction which is a critical risk factor for the DED occurrence (28, 29). We speculate that the persistent applications of coffee and tea could associate with less anxiety in our depression participants more prominently, while the depression and anxiety own dual effect concerning the development of DED (11). Further research should focus on this issue because exercise, coffee consumption and tea consumption are all simple and low-cost activities, and both depression and DED affect the majority of the population (2, 30).

Furthermore, when comparing the lifestyle and nutritional characteristics of patients with DED and depression and those without depression, only the incidence of persistent cigarette smoking was significantly higher in those with concurrent DED and depression. In previous smoking cessation studies, cigarette smoking has been proposed as a risk factor for DED development (8, 31). Our findings suggested that persistent cigarette smoking may significantly correlate with DED in general population but not in specific situations such as depression. Also, the incidence of persistent cigarette smoking in the depression with DED subgroup was higher than that in the depression without DED subgroup, although the difference was not significant. Interestingly, the persistent alcohol drinking and betelnut chewing occurrence did not reveal significant differences between the DED with depression and DED without depression subgroups; however, both lifestyle and nutritional factors were significantly higher in the depression without DED subgroup than in the depression with DED subgroup. This finding could lead to the misconception that persistent alcohol drinking and betelnut chewing are protective factors for DED in the depressed population, which conflicts with previous conceptions (2). Nevertheless, the insufficient number of cases of these two lifestyle and nutritional factors would lead to statistical bias, and further study is warranted to survey the relationship between cigarette smoking, betelnut chewing, and DED occurrence in more details.

Regarding lifestyle and nutritional characteristics between the two groups, individuals without a university education, unmarried or divorced, chronic pain, persistent cigarette smoking, and VDT users were significantly higher in the depression group than in the non-depression group. Low education level, unmarried status, and chronic pain are well-established risk factors for depressive disorders; thus, it is reasonable that these conditions were higher in the depression group in this study (19, 32, 33). Although the relationship between cigarette smoking and depression is inconsistent (34), persistent cigarette smoking may cause negative emotions and cognitive impairments in individuals (35). Our findings support a positive correlation between persistent cigarette smoking and depression. The reason for the higher percentage of VDT users in the depression group remains unclear; nonetheless, a possible explanation for this result is that occupations requiring frequent use of VDT are generally high-stress work and are likely to cause negative emotions and affective disorders (36).

This study had some limitations. First, since most of the questionnaires were only filled out by participants at the start of the exam, we could only conduct a retrospective case–control study, which limited the ability to search for a causal relationship between DED and depression. Second, all the information we obtained in this study, except for age and sex, was based on the questionnaire filled out by each participant. Consequently, the accuracy of diagnoses, the details of disease-related examinations, the severity of disease, the treatment and therapeutic outcome of each disease, the subtype of depression disorder, the use of eye drops that could lead to DED, and the amount of alcohol, cigarettes, and betelnut consumption could not be validated, which significantly influenced the outcome of our study. Additionally, most of the patients participated in the TWB project between 2012 and 2015, which was slightly outdated and may have caused some bias concerning the time of data collection. Moreover, a large number of approximately 15–20% of patients were lost because we excluded participants who did not answer the question we wanted. However, the influence of patient exclusion on statistical power might not be significant since this study population was over 10,000 participants.

In conclusion, chronic pain and sedentary lifestyle incidence were significantly higher in patients with depression and DED than in those without DED. Furthermore, persistent coffee and tea consumptions was more prevalent in patients with depression but without DED than those patients with concurrent depression and DED. Consequently, physical activity, coffee intake and tea intake may be encouraged in those with depression to reduce the risk of developing DED. Further large-scale prospective studies are required to survey whether the increment of physical activity, coffee consumption and tea consumption would alter the therapeutic outcome of DED in patients with depression.

## Data Availability

The raw data supporting the conclusions of this article will be made available by the authors, without undue reservation.
